# The Utility of Immune Checkpoint Inhibition in the Management of Resectable Non-Small Cell Lung Cancer

**DOI:** 10.3390/cancers17152462

**Published:** 2025-07-25

**Authors:** Louis Filipiak, Koosha Paydary, Mary Jo Fidler, Helen J. Ross

**Affiliations:** Department of Medicine, Section of Hematology, Medical Oncology and Cell Therapy, Rush University Medical Center, Chicago, IL 60612, USA; louis_g_filipiak@rush.edu (L.F.); mary_fidler@rush.edu (M.J.F.); helen_ross@rush.edu (H.J.R.)

**Keywords:** Non-small cell lung cancer, Immune checkpoint inhibitor, perioperative

## Abstract

Immune checkpoint inhibitors (ICIs) are approved in at least one line of therapy for most patients with advanced non-small cell lung cancer (NSCLC) without EGFR/ALK alterations and have improved survival for a subset of patients. Adjuvant, neoadjuvant, and perioperative therapy for resectable NSCLC carries the hope of more broadly increased cure rates for patients with resectable lung cancers. This review summarizes the current state of multimodality management, including ICIs, for resectable NSCLC. A literature search of PubMed and Scopus identified phase II and III clinical trials including ICIs in patients with resectable NSCLC. No level 1 evidence guides the clinician in choosing between the available neoadjuvant and perioperative approaches.

## Simple Summary

Immune checkpoint inhibitors (ICI) are a type of treatment that helps the body’s immune system recognize and attack cancer. They have already improved survival for many people with advanced, metastatic lung cancer. Recently, researchers have begun testing whether these treatments can also help patients whose lung cancer is still operable—by giving the therapy before or after surgery. This review explores recent studies of these treatments in combination with chemotherapy around the time of surgery. These studies suggest that adding immune therapy may improve the chances of curing the cancer and preventing it from coming back. However, there is no clear agreement yet on the best timing or combination strategy. The review also highlights important areas for future research, including how to choose the right patients for each treatment approach. These developments could help more people with lung cancer live longer and healthier lives.

## 1. Introduction

According to SEER epidemiologic data, lung cancer is the most common cause of cancer death in the USA [1]. Deaths from lung cancer continued at relatively static rates for decades until the recent development of targeted and immune-focused therapies, which have at last led to improving outcomes. Five-year survival of patients diagnosed with lung cancers has nearly doubled from 13.7% in 1992 to 26.7% in 2024 [1]. The first demonstration of survival benefit from immunotherapy in non-metastatic NSCLC was from the PACIFIC trial, which randomly assigned patients with unresectable stage III NSCLC (AJCC v 7), including those who had completed standard of care chemoradiotherapy with neither progression nor decline in performance status to durvalumab vs. placebo. The PACIFIC trial reported an absolute 17% progression-free survival advantage for patients receiving durvalumab compared to chemoradiotherapy alone in an 18-month span [2]. Subsequent studies exploring the efficacy of adjuvant ICIs in earlier stage disease are in progress or have been completed. In this paper, we will review published studies on the safety and efficacy of perioperative immunotherapy in NSCLC focusing on randomized phase III trials (Table 1). We performed a comprehensive literature search via PubMed using the search terms “neoadjuvant”, “adjuvant”, “perioperative”, “non-small cell lung cancer”, and “immune checkpoint inhibitor”. For practicality, we focused solely on phase III and selected phase II trials that evaluated the safety and efficacy of ICIs in resectable NSCLC.

## 2. Adjuvant Clinical Trials

### 2.1. IMpower 010

This phase III trial preceded studies of neoadjuvant combination therapy and was the first to demonstrate a survival advantage of adjuvant immunotherapy in the resectable NSCLC evaluated [7]. A total of 1005 patients with resected stage IB-IIIA (AJCC v 8) NSCLC were randomly assigned to the adjuvant atezolizumab for one year (507) or control (498) following standard adjuvant chemotherapy. The primary endpoint was DFS by investigator assessment in the stage II-IIIA population with PD-L1 at least 1% by the Ventana SP263 assay; secondary endpoints included OS and safety. This trial showed an overall survival advantage to adjuvant atezolizumab. DFS in the primary efficacy analysis population was 35.3 months (95% CI: 29.0, NE) for BSC vs. not reached (95% CI: 36.1, NE) for atezolizumab (HR 0.66; 95% CI: 0.50, 0.88; *p* = 0.004). In a pre-specified secondary subgroup analysis of patients with PD-L1 TC ≥ 50% stage II-IIIA NSCLC, the DFS HR was 0.43 (95% CI: 0.27, 0.68). In an exploratory subgroup analysis of patients with PD-L1 TC 1–49% stage II-IIIA NSCLC, the DFS HR was 0.87; (95% CI: 0.60, 1.26). At a median of 45.3 months, the median OS HR was higher in patients with higher PD-L1 TPS levels as follows: 0.95 (95% CI: 0.74–1.24) in overall population, 0.71 (95% CI: 0.43–1.03) in TPS >1%, and 0.43 (95% CI: 0.24–0.78) in TPS >50% [7]. Common grade 3 or higher treatment adverse related events in the treatment arm included rash (18.4%), hepatitis (17.6%), and hypothyroidism (17.0%). Crucially, atezolizumab-related adverse events did not differ significantly from their interim cutoff point in 2021, though a higher number of patients in the treatment group did experience at least grade 3 adverse events (22.0% vs. 11.5%) [7]. The findings of this study resulted in the FDA approval of adjuvant atezolizumab on October 15, 2021, for resected stage II-IIIA NSCLC for patients whose tumors’ PD-L1 TPS is >= 1%.

### 2.2. PEARLS/Keynote-091

In this phase III trial, the safety and efficacy of adjuvant pembrolizumab was assessed in 1177 patients with resected stage IB-IIIA (AJCC v 8) NSCLC (86% of whom in each arm had completed adjuvant platinum doublet chemotherapy), who were randomly assigned to adjuvant pembrolizumab or placebo [8]. A total of 590 patients in the experimental arm received post-resection pembrolizumab 200 mg every three weeks, while 587 patients in the control arm received adjuvant placebo. After a median follow up of 35.6 months, the primary endpoint of DFS favored the pembrolizumab group with DFS 53.6 months vs. 42.0 months in the control group (HR: 0.76, 95% CI: 0.63–0.91). Grade 3 or higher safety events occurred in 34% of the pembrolizumab group vs. 26% of the control group [8]. The FDA approved adjuvant pembrolizumab monotherapy in resectable NSCLC patients regardless of PD-L1 status on 26 January 2023 [8].

Notably, the most common forms of grade 3 or higher Treatment Related Adverse Events (TRAEs) in both adjuvant studies were rash, hepatitis, and hypo/hyperthyroidism, myocarditis and pneumonitis. [7,8].

## 3. Neoadjuvant Clinical Trials

### Checkmate-816

In this phase III study, 358 patients with stage IB-IIIA (AJCC v 8) resectable NSCLC and without EGFR/ALK alterations were randomly assigned to neoadjuvant nivolumab 360 mg every three weeks for three cycles (358 participants) along with platinum-based doublet chemotherapy vs. chemotherapy alone [4]. A third treatment arm with chemotherapy plus nivolumab and ipilimumab closed early -with the reported results from the NEOSTAR, NADIM, and Checkmate-9LA trials- which showed superiority with the combined regimen of nivolumab and ipilimumab over neoadjuvant nivolumab alone as well as superiority of the combined therapy to chemoradiotherapy. Event-Free Survival (EFS) and pathologic complete response (pCR) were the primary endpoints, and the secondary endpoint was OS. Median EFS was 31.6 months vs. 20.8 months, favoring nivolumab (HR: 0.63, 95% CI: 0.43–0.91), while pCR was 24.0% and 2.2% (95% CI: 3.49–55.75), respectively. A subgroup analysis suggested the largest trend to improved EFS and pCR was present for participants who were younger, female, or had the highest PD-L1 (TPS >= 50%) [4]. Resection was completed for 83.2% vs. 75.4% for nivolumab vs. placebo arms, respectively. OS was not significantly different at 82.7% vs. 70.6% (95% CI: 0.3–1.07) [4]. Neoadjuvant nivolumab with chemotherapy was approved by the FDA on 4 March 2022.

Additionally, a four-year update analysis of Checkmate-816 showed further improvement in EFS: 43.8 months in the treatment arm vs. 18.4 months in the control arm (HR: 0.66, 95% CI: 0.49–0.90), regardless of the type of surgical intervention [9]. Median 4-year OS also improved between groups with 71% OS in the treatment group and 58% in the control group (HR: 0.08. 95% CI: 0.02–0.34) [9]. No new safety signals were observed within the four-year update.

Notably, grade 3 or 4 TRAEs occurred in 33.5% of the treatment group compared to 36.9% in the control group [4]. While neutropenia was the most common side effect between both groups, the nivolumab arm was more likely to experience rashes (8.5% vs. 1.1%). Finally, two patients in the nivolumab arm experienced fatal adverse events precluding surgery [4].

## 4. Perioperative Clinical Trials

### 4.1. Keynote-671

This randomized, double-blind phase III trial of four cycles of neoadjuvant platinum-doublet chemotherapy with pembrolizumab vs. placebo followed by adjuvant pembrolizumab or placebo for up to 13 cycles enrolled participants with resectable stage IIA-IIIB NSCLC (AJCC v 8) [3]. The primary endpoints of the study were EFS and OS, with secondary endpoints including Major Pathological Response (MPR), pCR, and safety. A total of 797 participants were randomly assigned to the experimental group (397 participants) or the standard of care group (400 participants). EFS at 24 months was 62.4% in the pembrolizumab group vs. 40.6% in the placebo group (HR: 0.58, 95% CI: 0.46–0.72). OS at 36 months was not statistically significantly different at 71% and 66%, respectively, according to updated data [10]. For pembrolizumab, MPR and pCR were 30.2% and 18.1%, respectively. For placebo, MPR and pCR were 11.0% and 4.0, respectively. In addition, 44.9% of the pembrolizumab group and 37.3% of the placebo group had a grade 3 or higher TRAE. Resection was carried out in 82.1% of patients in the pembrolizumab group vs. 79.4% in the chemotherapy alone group [11]. More responses occurred in patients with PD-L1 TPS score >50%. Responses were the lowest for those with PD-L1 less than 1%. Participants with EGFR or ALK gene mutations noted on Next Generation Sequencing (NGS) had lower EFS than their wild-type counterparts [11]. Keynote-671 showed that a perioperative approach of chemoimmunotherapy prior to resection and adjuvant pembrolizumab significantly improved EFS, MPR, and pCR compared to neoadjuvant chemotherapy alone. Perioperative pembrolizumab (with neoadjuvant chemotherapy) was approved by the FDA on 16 October 2023.

### 4.2. Aegean

Similarly to Keynote 671, this phase III trial evaluated perioperative durvalumab in stages IIA-IIIB NSCLC (AJCC v 9) [4]. Participants were randomized to four cycles of platinum-based doublet chemotherapy with durvalumab every three weeks, followed by surgery and adjuvant durvalumab every four weeks for twelve cycles (401 participants). The control group of 398 patients received neoadjuvant chemotherapy plus placebo followed by adjuvant placebo [4]. The primary endpoints were EFS and pCR; safety was a secondary endpoint. Patients with EGFR/ALK alterations were excluded from the analysis, though several were initially included in the ITT group (51 for EGFR and 11 for ALK) due to poorer responses in several other studies. EFS at 24 months was 63.3% vs. 52.4% favoring the treatment arm (HR: 0.68, 95% CI: 0.53–0.88). pCR was 17.2% and 4.3% for durvalumab vs. placebo. Grade 3 or4 AEs occurred in 42.4% (durvalumab) and 43.2% (placebo) of patients [4]. Resection rates were 80.6% and 80.7%, respectively. Durvalumab with platinum-based chemotherapy prior to resection and durvalumab following resection was approved by the FDA on 15 August 2024.

### 4.3. Neotorch

This phase III trial conducted in China randomized 501 patients with stage II-III NSCLC (AJCC v 9) to platinum-based doublet chemotherapy plus the PD-L1 antibody toripalimab (202 participants) or placebo (202 participants) group. The vast majority of participants were male (92%) and 77.7% of patients enrolled had squamous cell histology [5]. Participants received a platinum-doublet plus neoadjuvant toripalimab 240 mg or placebo every three weeks for three cycles and adjuvant toripalimab or placebo every three weeks for 13 cycles. Primary endpoints were EFS and MPR. Secondary endpoints were OS, pCR, and Disease-Free Survival (DFS) [5]. After a median follow-up of 18.3 months, EFS was not reached in the treatment group vs. 15.1 months in the control group (HR: 0.4, 95% CI: 0.28–0.57). The MPR and pCR were 48.5% and 24.8% vs. 8.4% and 1% in the toripalimab and control groups, respectively. Median OS was not estimable in the toripalimab group and 30.4 months in the placebo group (HR 0.62, 95% CI: 0.38–1.00). Higher PD-L1 levels correlated with better outcomes (EFS HR 0.65, 95% CI: 0.33–1.23; HR of 0.31, (0.17–0.54); and HR of 0.3, (0.15–0.6) for PD-L1 TPS levels of <1%, 1–49% and >50%, respectively [5]. Resection rates were 82.2% for toripalimab vs. 73.3% for placebo [5]. Finally, grade 3 or 4 TRAEs were 63.4% in the toripalimab groups vs. 54.0% in the control group.

### 4.4. Checkmate-77T

In this randomized phase III trial, 461 patients with stages IIA-IIIB NSCLC (AJCC v 9) were randomly assigned to neoadjuvant platinum-doublet plus nivolumab (229) or placebo (232) every three weeks for four cycles followed by resection and adjuvant nivolumab or placebo every four weeks for one year. The primary endpoint was EFS, and the secondary endpoints were pCR, MPR, and OS [12]. At 25.3 months follow up, EFS was 70.2% in the treatment group and 50.0% for the control group (HR: 0.58; 95% CI: 0.42–0.81) and pCR was 25.3% vs. 4.7% (HR: 6.64; 95% CI: 3.40–12.97). [6]. Grade 3 or higher adverse events were not statistically significantly different at 32.5% compared to 25.2% for nivolumab vs. placebo. Resection rates were 78% vs. 77% [6]. Neoadjuvant nivolumab and platinum doublet chemotherapy followed by adjuvant nivolumab was FDA approved for eligible patients on 3 October 2024.

Regarding safety, patients in the perioperative arms of these respective studies were either equally or more likely to experience grade 3 or 4 TRAEs than the control arms. The most common types of TRAEs included neutropenia, anemia, thrombocytopenia, rashes, and hyper/hypothyroidism. Fatal grade 5 events in the treatment arms of these perioperative studies included pneumonitis, cerebral infarction, and sudden cardiac death spurned by atrial fibrillation [4,5,11].

## 5. Other Perioperative Approaches Under Study

The studies discussed above focused on the utility of ICIs with or without chemotherapy. The safety and efficacy of several other approaches are under study in earlier phase trials. The NEOpredict-Lung study, whose primary endpoint was feasibility of surgery at six weeks, has shown encouraging 2-year OS and DFS with a short course of neoadjuvant nivolumab plus relatlimab (LAG-3 inhibitor) in a chemotherapy-free regimen [11,13]. The global phase II multi-platform NeoCOAST-2 study combined neoadjuvant chemotherapy with durvalumab and other novel anticancer agents such as oleclumab (anti-CD73 mAb), monalizumab (anti-NKG2AmAb), or datopotamab deruxtecan (Dato-Dxd, TROP2-directed antibody drug conjugate (ADC)) [14]. This study provided a treatment group alone, with four cycles of neoadjuvant durvalumab with each of the respective agents, and it measured pCR, MPR, the rate of patients that underwent surgery, and the rate of patients that underwent adjuvant therapy. The preliminary results showed higher pCR (34.1% vs. 26.7% vs. 20.0%, respectively) and MPR (65.9% vs. 53.3% vs. 45.0%, respectively) for Dato-DxD than monalizumab or oleclumab, pointing to encouraging efficacy for antibody–drug conjugates in the neoadjuvant setting [15].

## 6. Discussion

The FDA has now approved ICIs in the perioperative, neoadjuvant, and adjuvant settings for resectable NSCLC based on the suggestion of improved outcomes for patients with resectable NSCLC. As of early 2025, perioperative pembrolizumab, perioperative durvalumab, perioperative nivolumab, neoadjuvant nivolumab, adjuvant atezolizumab (in patients with PD-L1 scores >= 1%), and adjuvant pembrolizumab have received FDA approvals. The best strategies for choosing between the available options are being explored in both retrospective and prospective analyses. The best perioperative strategy remains unclear in the absence of randomized data comparing neoadjuvant to perioperative approaches. In fact, heterogeneity among studies is modest, with a mean I^2^ statistic (which measures the percent variation in studies due to heterogeneity rather than chance) of 42%. In other words, physicians can currently view these regimens as interchangeable and can weigh regimens on other factors such as cost or individual preference [15]. Guidance for patients with stage II NSCLC is particularly important considering the smaller numbers of stage II patients enrolled in published studies of neoadjuvant and perioperative ICIs. Greater enthusiasm for surgery for patients with more advanced stage III NSCLC and paucity of data on multi-station N2 disease also requires greater guidance for those patients.

Notably, data pooled from the perioperative Keynote-671, Aegean, Checkmate-77T, and Neotorch trials did show a small survival benefit in patients with a PD-L1 < 1% with a hazard ratio for risk of progression or death of 0.73 (95% CI: 0.6–0.89), implying a benefit to perioperative rather than neoadjuvant therapy alone. Furthermore, pooled data from these same trials also showed a clear advantage for at least neoadjuvant ICI use in resectable stage II patients with a hazard ratio for risk of progression and death of 0.64 (95% CI: 0.49–0.82) [15]. Nonetheless, such analysis from pooled data of several trials could be subject to bias and therefore should be interpreted with caution as prospective data are lacking.

### 6.1. Choosing from the Available Perioperative Options

There is as yet no consensus regarding the best neoadjuvant or perioperative option for patients with resectable NSCLC. The studies discussed in this review have used different measures of response and included patients with different demographic and clinical features. As always, cross-trial comparisons should be avoided. Among the unknowns is the proportion of benefit from each phase of treatment. An exploratory propensity score weighted analysis of the two trials using nivolumab—Checkmate 77T (limited to only patients who received at least one dose of adjuvant nivolumab) and Checkmate 816—favored a perioperative approach, supporting further benefit driven from the continuation of adjuvant nivolumab, although this can only be considered hypothesis generating [16]. The benefit of adjuvant nivolumab appeared more pronounced among patients who had not achieved pCR following the adjuvant chemo-nivolumab, although again this is a speculative finding. Perhaps the effective priming of the immune system preoperatively and continued suppression of PD-L1 mediated immune inhibition as well as the elimination of micro-metastatic disease could lead to improved OS with the perioperative approach compared to neoadjuvant or adjuvant only approaches [17]. While about 15–20% of surgeries may be canceled due to tumor progression or complications and toxicities of neoadjuvant treatment, the rate of resections was not different in the presence or absence of ICIs.

### 6.2. Utility of Pathological Response Rates

The utility of pCR and MPR as surrogate endpoints for EFS and OS was explored in a recent meta-analysis of neoadjuvant ICI trials by Hines et al. A robust correlation was observed between pCR/MPR and 2-year EFS, but not with OS [18]. The lack of correlation between pCR/MPR and OS remains unexplained but may be due to treatment crossover and immaturity of the OS data. With respect to the duration of neoadjuvant phase and its correlation with pCR, the pCR was reported as 24%, 18%, and 25.3% in Checkmate-816 (3 cycles of chemo-nivolumab), Keynote-617 (4 cycles of chemo-pembrolizumab), and Checkmate-77T (4 cycles of chemo-nivolumab), respectively. Therefore, it does not appear that increasing the number of cycles of neoadjuvant cycles of chemoimmunotherapy from 3 to 4 is likely to be associated with increased pCR. Whether fewer cycles would achieve the same results has not been studied, nor has the effect of the histology, stage, nodal involvement, or distribution of PD-L1 among the study populations. Using pCR/MPR as a primary endpoint offers the advantage of fast results; however, the clinical utility of these measures in a real-world setting and their correlation with the number of treatment cycles is controversial. Initiatives such as the International Association for the Study of Lung Cancer (IASLC) Pathologic Response Project may shed light on the utility of pCR as a surrogate endpoint for survival and may identify patient subgroups that could benefit from shorter treatment courses [19,20].

Another challenge of the multimodality management of stage III NSCLC is the possible impact on R0 resections. While about 80–90% of the patients enrolled in neoadjuvant trials underwent surgery, investigators agree that incomplete resections likely do not confer a survival benefit. Expert consensus remains that the decision to pursue surgery should be made before starting neoadjuvant treatment. The definition of resectability may vary by multidisciplinary strengths and institutional factors, and the role of perioperative approaches for borderline/potentially resectable patients is unknown. Potential delays in surgical resection and the increased difficulty of surgery after neoadjuvant ICIs are common topics of multidisciplinary discussion. No delay in surgery was observed in the majority of perioperative trials, and overall, it appears that the neoadjuvant phase does not alter the choice of surgical procedure in the highly selected study populations [4,11].

Ongoing and future trials should assess the nature and duration of adjuvant therapy after resection following neoadjuvant treatment. For example, it is unclear whether adjuvant pembrolizumab should be continued in patients who achieved pCR following neoadjuvant treatment. A post hoc analysis of EFS in Keynote-671 reported that that lower percentage of residual viable tumor (RVT) was associated with improved EFS [21]. In this analysis, pathological regression measured as a percentage of RVT < 60% may be a viable surrogate endpoint.

### 6.3. Predictive Biomarkers of Response to Immunotherapy

The identification of predictors of improved response to immunotherapy in resectable NSCLC is an area of active research. In general, it appears that younger patients and those with a smoking history and higher PD-L1 TPS are associated with a higher degree of response [4,5,7,8,9,11,12]. Nonetheless, these speculations are based on the subset analysis of patients among various trials and lack prospective validation. With respect to PD-L1, the benefit conferred to patients with negative PD-1 expression, i.e., TPS < 1%, should be further investigated. Generally, tumors with greater PD-L1 staining show greater pathological responses [22]. Consequently, the potential role of PD-L1 as a biomarker in at least neoadjuvant chemoimmunotherapy remains to be validated in larger prospective studies [23]. Additionally, among studies that included patients with EGFR and ALK alterations—such as Keynote-671, Aegean, and IMpower 010—the use of ICIs did not result in improved outcomes among EGFR/ALK altered subsets [4,7,9]. In light of these observations, NCCN guidelines recommend that patients that have EGFR or ALK alteration should not receive neoadjuvant immunotherapy, underscoring the importance of molecular profiling prior to decisions about pre/peri-operative chemoimmunotherapy. Other notable biomarkers that are currently being reviewed as developing prospects include tumor-infiltrating immune cells (TILs) and tumor mutational burden (TMB) [23].

### 6.4. Financial Toxicity and Patient Convenience

While the incorporation of ICIs in the multimodality management of resectable NSCLC has become a standard of care for many patients, other factors including financial and time toxicity and increased risk of immune-related adverse events may influence patient experience. A review of data from the Keynote-671 study showed a cost of 53,637 USD per Quality-Adjusted Life Year (QALY) for the treatment group versus 37,046 USD per QALY for the control group [24]. Ongoing investigations into the financial, time, and clinical toxicities of neoadjuvant/perioperative ICIs will help inform treatment choices. In an indirect meta-analysis of several perioperative approaches, the incidence of any grade TRAEs significantly increased with the addition of adjuvant immunotherapy, and the adjuvant phase increased grade 3 or greater toxicities by 4–10% [25]. Several other factors such as the time spent traveling to the healthcare facility, parking payment, co-pays, and the necessity to take time off for patients and caregivers can substantially influence patients’ experience when an adjuvant or perioperative approach is planned. Perioperative ICI trials should integrate patient-centered outcomes to help guide treatment choices.

A suggested trial design by Chaft et al. could address questions of optimal cycle length and toxicities [26]. It is proposed that a theoretical study could consist of the following four arms: a neoadjuvant novel drug plus standard-of-care (SOC) followed by surgery and adjuvant SOC, a neoadjuvant SOC followed by surgery and an adjuvant novel drug plus SOC, a neoadjuvant novel drug plus SOC followed by surgery and the adjuvant novel drug plus SOC, and a control group receiving neoadjuvant SOC followed by surgery and adjuvant SOC [27]. In this way, the efficacy of each phase of treatment could be determined.

## 7. Conclusions

Clinical trials of neoadjuvant ICIs have changed the standard management of resectable NSCLC for ICI-eligible patients. The neoadjuvant and perioperative use of ICIs for resectable NSCLC carries the hope of more broadly increased cure rates for patients with resectable lung cancers. Patient selection is of utmost importance, and multidisciplinary discussion is vital to appropriately select patients. Patient selection may be particularly challenging in the community setting where surgical expertise to address the increased inflammation due to immune infiltration within the tumor and involved lymph nodes is important. It is noteworthy that while the vast majority of patients with resectable NSCLC are managed in the community setting, it is not known to what degree perioperative approaches are implemented. The increased inflammation from neoadjuvant ICI can be a potential operative challenge and thus surgical expertise can be an essential factor in achieving optimal surgical outcomes, though meta-analyses do show that ICI use is safe from a surgical perspective [28]. Moreover, NGS is a must as EGFR/ALK alterations should be ruled out prior to the start of neoadjuvant ICIs. Further unanswered questions include the optimal number of cycles of treatment in each phase, the correlation between pCR/MPR with OS, role for and type of additional therapy in the absence of MPR/pCR, and optimal salvage approach in patients with disease progression following neoadjuvant chemoimmunotherapy [29]. Future trials will continue to evaluate the extent of benefit and safety of perioperative ICI use in resectable NSCLC.

## Figures and Tables

**Table 1:** Major phase III clinical trials of immune-checkpoint inhibitor use in resectable non-small cell lung cancer.

Trial	# of Patients	Treatment Arm	Control Arm	Stages Included	EGFR/ALK Included?	% of Patients That Underwent Surgery in Both Arms (ICI vs. Placebo)	Efficacy Results	Percent of Patients with PD-L1 >= 1%
Keynote-671 [3]	797	Perioperative pembrolizumab with four cycles of neoadjuvant pembrolizumab plus cisplatin-based doublet chemotherapy	Neoadjuvant cisplatin-based doublet chemotherapy alone with adjuvant placebo	IIA-IIIB	Yes-54 patients	82.1% vs. 79.4%	EFS HR: 0.58 *; OS HR: 0.73	64%
Aegean [4]	802	Perioperative durvalumab with four cycles of neoadjuvant durvalumab plus platinum-based doublet chemotherapy	Neoadjuvant platinum-based doublet chemotherapy alone with adjuvant placebo	IIA-IIIB	No	80.6% vs. 80.7%	EFS HR: 0.68 * pCR OR: 4.62 *	66.7%
Neotorch [5]	501	Perioperative toripalimab with three cycles of neoadjuvant toripalimab plus platinum-based doublet chemotherapy	Neoadjuvant platinum-based doublet chemotherapy alone with adjuvant placebo	IIA-IIIB	No	82.2% vs. 73.3%	EFS HR: 0.4 *; MPR OR: 10.9 *	65.6%
CheckMate 77-T [6]	461	Perioperative nivolumab with four cycles of neoadjuvant nivolumab plus platinum-based doublet chemotherapy	Neoadjuvant platinum-based doublet chemotherapy alone with adjuvant placebo	IIA-IIIB	No	77.7% vs. 76.7%	EFS HR: 0.58 *	55.5%
CheckMate-816 [4]	358	Neoadjuvant nivolumab for three cycles plus platinum-based doublet chemotherapy	Neoadjuvant platinum-based doublet chemotherapy alone	IB-IIIA	No	83.2% vs. 75.4%	EFS HR: 0.63 *; pCR OR: 13.94 *	49.7%
Impower-010 [7]	1005	Adjuvant atezolizumab with cisplatin-based doublet chemotherapy	Adjuvant cisplatin-based doublet chemotherapy alone	IB-IIIA	Yes-20 patients	100%	DFS with TPS > 1% HR: 0.66 *; DFS with TPS > 50% HR: 0.43 *	54.6%
PEARLS/Keynote-091 [8]	1177	Adjuvant pembrolizumab monotherapy	Adjuvant placebo alone	IB-IIIA	Yes-39 patients	100%	DFS HR: 0.73 *	60.5%

(TPS: tumor proportion score; EFS: event-free survival; OS: overall survival; pCR: pathological complete response; MPR: major pathological response; DFS: disease-free survival; ICI: immune checkpoint inhibitor). * Indicates statistical significance.
